# Immune Response of the Monocytic Cell Line THP-1 Against Six *Aeromonas* spp.

**DOI:** 10.3389/fimmu.2022.875689

**Published:** 2022-07-08

**Authors:** Ana Fernández-Bravo, Maria José Figueras

**Affiliations:** ^1^ Rovira i Virgili University, Department of Basic Medical Sciences, Mycology and Environmental Microbiology Unit, Reus, Spain; ^2^ Pere Virgili Health Research Institute (IISPV), Reus, Spain

**Keywords:** *Aeromonas* spp., immune-related genes, monocytic cells, cell damage, intracellular survival

## Abstract

*Aeromonas* are autochthonous bacteria of aquatic environments that are considered to be emerging pathogens to humans, producing diarrhea, bacteremia, and wound infections. Genetic identification shows that 95.4% of the strains associated with clinical cases correspond to the species *Aeromonas caviae* (37.26%), *Aeromonas dhakensis* (23.49%), *Aeromonas veronii* (21.54%), and *Aeromonas hydrophila* (13.07%). However, few studies have investigated the human immune response against some *Aeromonas* spp. such as *A. hydrophila*, *Aeromonas salmonicida*, and *A. veronii*. The present study aimed to increase the knowledge about the innate human immune response against six *Aeromonas* species, using, for the first time, an *in vitro* infection model with the monocytic human cell line THP-1, and to evaluate the intracellular survival, the cell damage, and the expression of 11 immune-related genes (*TLR4*, *TNF-α*, *CCL2*, *CCL20*, *JUN*, *RELA*, *BAX*, *TP53*, *CASP3*, *NLRP3*, and *IL-1β*). Transcriptional analysis showed an upregulated expression of a variety of the monocytic immune-related genes, with a variable response depending upon the *Aeromonas* species. The species that produced the highest cell damage, independently of the strain origin, coincidentally induced a higher expression of immune-related genes and corresponded to the more prevalent clinical species *A. dhakensis*, *A. veronii*, and *A. caviae*. Additionally, monocytic cells showed an overexpression of the apoptotic and pyroptotic genes involved in cell death after *A. dhakensis*, *A. caviae*, and *Aeromonas media* infection. However, the apoptosis route seemed to be the only way of producing cell damage and death in the case of the species *Aeromonas piscicola* and *Aeromonas jandaei*, while *A. veronii* apparently only used the pyroptosis route.

## Introduction

The genus *Aeromonas* comprises species considered autochthonous of aquatic environments that can also cause a wide spectrum of diseases in humans, mainly gastroenteritis, bacteremia/septicemia, and wound infections. *Aeromonas* are considered emerging pathogens due to the increase of their isolation as well as becoming renowned as a pathogen of serious public health concern (1–5). The infection occurs more frequently in children, the elderly, and immunocompromised individuals, and cases of bacteremia with fever, jaundice, abdominal pain or septic shock, and extraintestinal infections involving meningitis, pneumonia, keratitis, and osteomyelitis among others (1, 2, 4, 6, 7). *Aeromonas* also were the microorganisms more isolated from wound infections after natural disasters like the tsunami in Thailand (2004), representing about 22.6% of all isolates in the survivors (8).

According to a recent review, 95.4% of the strains associated with clinical cases correspond to four species: *Aeromonas caviae* (37.26%), *Aeromonas dhakensis* (23.49%), *Aeromonas veronii* (21.54%), and *Aeromonas hydrophila* (13.07%) (5). However, other species, i.e., *Aeromonas media* (2.27%) and *Aeromonas jandaei* (0.43%), have also been involved with a lower frequency. The virulence of *Aeromonas* spp. has been considered multifactorial and associated with different toxins—aerolysin, hemolysin, lipases, and enterotoxins—some of them delivered by different types of secretion systems as well as produced through a signal molecules such as *N*-acyl homoserine-lactone (AHLs) by a phenomenon defined as quorum sensing, which results in the colonization, invasion, and proliferation of the bacteria during the infectious process (9–12). However, the development of the infection depends on the immunity of the host, which is divided into two types: innate and adaptive (13). The innate immune response, that is, the one investigated in the present study, is activated after recognition of bacteria structures, i.e., pathogen-associated molecular patterns (PAMPs) by receptors named pattern recognition receptors (PRRs), soluble and insoluble, present and expressed in a variety of immune-related cells, like neutrophils, monocytes, or macrophages (13–16). Toll-like receptors (TLRs) are membrane PRRs that induce the phagocytosis of the pathogen and activate the expression of cytokines in the host, initiating the inflammatory response (17). It has been demonstrated that TLR recognition such as TLR2, TLR3, or TLR4, depending on the organisms, induces cell death by apoptosis (18). Apoptosis is a caspase-dependent process that induces nuclear condensation and the release of cytoplasmic content from the host cell into the extracellular environment, which prevents inflammation (19). Moreover, other types of PRRs named the Nod-like receptors (NLRs) are expressed in the cytosol and induce a different type of cell death called pyroptosis, mediated by the activation of caspase-1 and by the formation of multi-protein complexes called the inflammasomes (14, 20). Pyroptosis can take place in many cell types, including macrophages, dendritic cells, neutrophils, and epithelial cells. This process is characterized by the activation of different types of** **caspase enzymes and contributes to the activation of pro-inflammatory cytokines, recruits more immune cells, and finally activates an inflammatory cascade in the tissue (14). In the last years, few studies have investigated the expression of several innate immune-related genes against *A. hydrophila*, *A. veronii*, and *Aeromonas salmonicida* in different cell lines or animal models (mice and fish) (12, 21–24). The majority of the studies has been performed with cell lines obtained from animals, with the exception of two cell lines (T-84 and WLR-68) obtained from the human colon (12, 24). However, human immune-related cell lines have, so far, not been used as cell models in the *Aeromonas* studies.

The upregulation of the TLR4, which is a transmembrane receptor that senses molecules such as the lipopolysaccharide (LPS) present in the cell walls of Gram-negative bacteria, has been detected in several studies after infecting a fish model with different strains of *A. hydrophila* (21–23, 25, 26). The TLRs also induce the activation of the nuclear factor NF-Kappa-B p65 subunit (RELA) protein, which is a protein involved with other proteins such as RELB, REL, p105/p50 NF-κB1 and NF-κB2 in the formation of the nuclear factor kappa-light-chain-enhancer (NF-κB) (17). For instance, after an *A. hydrophila* fish infection, the RELA protein was activated by the upregulation of the expression levels of the *TLR3*, *TLR4-1*, *TLR9*, and *TLR22* genes and inducing the production of pro-inflammatory cytokines (27). A previous study indicated that changes in the expression of the *JUN* proto-oncogene, which is another transcription factor that binds with the *FOS* proto-oncogene, could be involved in the virulence of *Aeromonas* spp. in mice because pathogenic strains induced upregulation of *JUN* and *FOS* genes (28). Both genes form a complex resulting in the formation of the activator protein-1 (AP-1), which is a transcription factor family implicated in critical cell functions such as the inflammatory response (29). Moreover, after *A. hydrophila* and *A. salmonicida* infections in fish, the activation of the gene expression that encodes pro-inflammatory cytokines like TNF-α and interleukin 8 (IL-8), as well as chemokines, i.e., the C-C motif ligand 20 (CCL20) involved in the recruitment of lymphocytes and dendritic cells, was demonstrated (21, 23, 30, 31). However, the C-C motif chemokine ligand 2 (CCL2) has not been studied in *Aeromonas*, despite it being an interesting chemokine well studied in infections produced by other bacteria like *Vibrio* spp., where it plays a role in the recruitment of monocytes, T cells such as CD4+ and CD8+, and dendritic cells (32). Therefore, the chemokine CCL2 will be investigated as a novel work. In relation to apoptosis, few studies demonstrated the capacity of *A. hydrophila*, *A. salmonicida*, and *A. veronii* to induce this process in different cell lines, and a fish infection model has been reported (25, 26). The caspase 3 (CASP3) protein is a member of the cysteine-aspartic acid protease family related to apoptosis and is known as executioner caspase by coordinating the destruction of cellular structures such as DNA fragmentation (19). In a previous study, it was observed that this gene was expressed during the apoptosis of head kidney-derived macrophages from fish infected with *A. hydrophila* (33). Another gene associated with apoptosis in *Aeromonas* is the tumor protein P53 (TP53) as Lü et al. (34) demonstrated after infecting a fish model with *A. hydrophila*. Previous studies demonstrated that TP53 induces the activation of the BCL-2-associated X protein (BAX), which belongs to the BCL-2 family, which plays an important role in the apoptosis route by the intrinsic pathway (27). However, the expression of the gene encoding the BAX protein has not been studied in *Aeromonas* and will be also investigated in the current study. As described above, an alternative way to the cell death produced by apoptosis is pyroptosis, which involves the formation of the inflammasome (14, 35). The activation of the pyroptosis route has been demonstrated by studying two proteins associated with the inflammasome, i.e., pyrin domain containing 3 (NLRP3) and IL-1β, after infections in mice and murine macrophages with *A. hydrophila* and *A. veronii*, respectively (36, 37).

So far, in *Aeromonas*, the immune response studies have not yet used the human monocytic cell line (THP-1), which was used successfully in studies for the *in vitro* infections to investigate the host–pathogen interactions in *Vibrio vulnificus* (32, 35). Considering the latter and the fact that human monocytes act as the first line of defense at the beginning of the infection process (38), the THP-1 cells were selected as the host in the present study. Moreover, no information exists so far about the immune response generated against *Aeromonas* species, which are frequently isolated in clinical cases such as *A. dhakensis* and *A. caviae.* Additionally, whether the capacity to develop an innate-immune response in the host cells is equal to the environmental and clinical strains is another aspect that has never been explored. Therefore, this study investigates *Aeromonas* species that show different frequencies of occurrence in clinical cases, using genetically identified strains of clinical and environmental origin in order to clarify if there exists a species-specific immune response that could explain their differential prevalence in human infections.

## Materials and Methods

### Bacterial Strains

The study was performed with 24 strains (10 clinical and 14 environmental) of six different species of which *A. dhakensis*, *A. media*, *A. jandaei*, *Aeromonas piscicola*, and *A. caviae* have not been studied until now, being the only exception *A. veronii* (**Table 1**). The clinical strains were isolated from feces, sputum, and wound human infections, while the environmental strains were isolated from sick fish and water. All strains came from a collection that was maintained in Tryptone Soya Broth (TSB) (Becton Dickinson GmbH, Heidelberg, Germany) plus glycerol (20%) at −80°C, and from there, they were grown in Tryptone Soya Agar (TSA) (Becton Dickinson GmbH, Germany) at 37°C for 24 h (similar growth rates). Their identity was previously determined based on the housekeeping gene sequencing such as *rpoD* (range between 498 and 596 bp) or *gyrB* (range between 413 and 523 bp) using primers and conditions previously described (45). Prior to infection, bacteria were regrown at 37°C in serum-free Dulbecco’s Modified Eagle’s Medium (DMEM; PAA Laboratories GmbH, Munich, Germany) under shaking conditions (100 rpm) for 18 h (32).

**Table 1:** Strains used in the study.

Strain	Origin	Reference
*Aeromonas dhakensis* CECT 5744^T^ GMV 704 128177 28B	Child feces with diarrhea Cetacean infection (ulceration of skin) Human feces Human wound infection	(11) This study This study This study
*Aeromonas caviae* CECT 838^T^ ESV-378 E01980 D50233	Guinea pig Fish gills Human feces Human feces	(39) (40) This study This study
*Aeromonas veronii* CECT 4257^T^ 123384 AE6 01.1	Human sputum Human feces Lake water Sick fish	(41) This study This study This study
*Aeromonas media* CECT 4232^T^ ESV-382 32679 ESV-360	Fisheries water Fish gills Human feces Fish kidney	(42) (40) This study (40)
*Aeromonas jandaei* CECT 4228^T^ 4300E CECT 4813 AE214	Human feces Lake water Human feces Lake water	(43) This study This study This study
*Aeromonas piscicola* CECT 7443^T^ AE169 CECT 7444 AE71	Sick fish Baltic Sea Rainbow trout Lake water	(44) This study (11) This study

### Virulence Gene Detection

The presence of different virulence-associated genes such as aerolysin (*aerA*), hemolysin (*hlyA*), cytotoxic enterotoxin (*act*), cytotonic enterotoxins (*ast* and *alt*), flagellin A (*flaA*) gene, Type III secretion system genes (*ascF* and *ascV*), and shiga toxin (*stx1*) was evaluated by PCR using specific primers and PCR conditions described in previous studies (46, 47).

### Cell Lines and Conditions

The human monocytic cell line THP-1 (48) was selected for the experiments because it is a valuable model for studying the innate immune response against bacteria. This cell line was maintained as a cell suspension in Roswell Park Memorial Institute Medium (RPMI-1640, PAA Laboratories GmbH) supplemented with 10% fetal bovine serum (FBS; PAA Laboratories GmbH, Munich, Germany) plus 1% penicillin–streptomycin (P/S) solution (PAA Laboratories GmbH, Munich, Germany) at 37°C and 5% CO_2_ (32). Before the infection experiments, cells were seeded in tissue culture plates containing DMEM without FBS and P/S at a concentration of 0.5 × 10^6^ cells/ml to obtain 1 × 10^6^ cells/ml after 3 h (32, 35).

### Infection

Cell line THP-1 was infected with each of the 24 *Aeromonas* strains (**Table 1**) using overnight cultures (18 h) in DMEM without FBS and P/S, at a multiplicity of infection (MOI) of 10 and 20, i.e., the ratio between the number of bacteria and the number of cells targeted (49). The control strain of *V. vulnificus* (CECT 4999) was used at MOI 5, as done in a previous study (32). The cultures were incubated at 37°C and 5% CO_2_.

### Intracellular Survival

Infected monocytes at MOI 10 and 20 (initial dose) were incubated at 37°C with 5% CO_2_ for 1 h, followed by gentamicin treatment (50 µg/ml) for 1 h to kill extracellular bacteria (time 0), and then the number of bacteria inside the monocytes was determined (50). Incubation of infected monocytes continued with the fresh DMEM and maintenance dose of gentamicin (2 µg/ml) for an additional 4 h. After this, the number of bacteria inside THP-1 was counted. Percent survival was calculated using the number of bacteria after 4 h of incubation and after gentamicin treatment (50 µg/ml) (50). Results were expressed as the average of the results obtained for all the strains of the same species.

### Cell Damage Assay (Lactate Dehydrogenase Assay)

After the infection at MOI 10 and 20, supernatants were obtained at different times (t = 3, 4, 5, and 6 h). Cell damage was determined by quantifying the lactate dehydrogenase (LDH) enzyme released into the culture media (supernatants), by using the Cytox 96 Non-Radioactive Cytotoxicity Assay (Promega, Madison, WI, USA), as described in the manufacturer’s instructions. To perform a standard curve, a bovine recombinant LDH (Sigma-Aldrich, St. Louis, MO, USA) was used, and the LDH levels of the samples were extrapolated from the curve (32). Results were expressed as the average of the results obtained for the clinical and environmental strains.

### Analysis of the Expression of the Genes Related to the Immune System

Eleven different genes implicated in the immune response against pathogens were selected to quantify their transcription levels by THP-1 cells in response to the infections produced with the different strains of the *Aeromonas* spp. in relation to the non-infected cells. The primers used to evaluate the expression of the selected genes were those from Murciano et al. (32) and Zhao et al. (51) and are listed in **Table 2**. The selected genes were those that encode for TLR4, cytokines, and chemokines (TNF-α, CCL2, and CCL20), apoptosis (TP53, BAX, and CASP3), and pyroptosis (NLRP3 and IL-1β) as well as genes of the transcription factors (JUN and RELA). After 4 h of infection at MOI 20, THP-1 cells were washed twice with PBS, and the RNA was isolated from the samples by using the GenElute™ Mammalian Total RNA Miniprep Kit (Sigma-Aldrich). RNA quality and integrity were confirmed spectrophotometrically using NanoDrop 2000, calculating the 260/280 and 260/230 ratios. The cDNA was transcribed from total RNA by using the iScript cDNA Synthesis Kit (Bio-Rad Laboratories, Inc., Hercules, CA, USA). A real-time PCR was performed with cDNA for quantification by using the Power SYBR^®^ green PCR Mastermix (Applied Biosystems^®^, Life Technologies, Glasgow, UK) on a StepOnePlus™ Real-Time PCR System (Applied Biosystems^®^). Threshold cycle (CT) values were obtained to establish the relative RNA levels of the tested genes, using the glyceraldehyde-3-phosphate dehydrogenase (*GAPDH*) gene as a housekeeping gene of reference. The relative gene expression was determined by using the delta-delta Ct (2^−ΔΔCt^) method that relays the signal from the real-time PCR, as done in a previous study (32). Results corresponded to the average of the results obtained for all the strains of the same species and were expressed as fold changes in relation to the non-infected cells.

**Table 2:** Primers used to target gene expression (32) (51).

Gene	Sequence (5′–3′)
***GAPDH***	Forward CATGAGAAGTATGACAACAGCCT Reverse AGTCCTTCCACGATACCAAAGT
***TLR4***	Forward AGTTGATCTACCAAGCCTTGAGT Reverse GCTGGTTGTCCCAAAATCACTTT
***JUN***	Forward TGCCTCCAAGTGCCGAAAAA Reverse TGACTTTCTGTTTAAGCTGTGCC
***RELA***	Forward ATGTGGAGATCATTGAGCAGC Reverse CCTGGTCCTGTGTAGCCATT
***TNF-*** α	Forward GAGGCCAAGCCCTGGTATG Reverse CGGGCCGATTGATCTCAGC
***CCL2***	Forward CCCCAGTCACCTGCTGTTAT Reverse TGGAATCCTGAACCCACTTC
***CCL20***	Forward GCAAGCAACTTTGACTGCT Reverse ATTTGCGCACACAGACAACT
***CASP3***	Forward GAAATTGTGGAATTGATGCGTGA Reverse CTACAACGATCCCCTCTGAAAAA
***BAX***	Forward CCCGAGAGGTCTTTTTCCGAG Reverse CCAGCCCATGATGGTTCTGAT
***TP53***	Forward CAGCACATGACGGAGGTTGT Reverse TCATCCAAATACTCCACACGC
***NLRP3***	Forward CGTGAGTCCCATTAAGATGGAGT Reverse CCCGACAGTGGATATAGAACAGA
***IL-1B***	Forward TTCGACACATGGGATAACGAGG Reverse TTTTTGCTGTGAGTCCCGGAG

### Analysis of the Expression of *aerA* and *act* Genes

Two different genes implicated in the virulence, *aerA* and *act*, were studied after THP-1 infections with one strain of each *Aeromonas* species, with the exception of *aerA* with *A. jandaei* infection due to the lack of this gene in all strains. The primers used to evaluate the expression of the selected genes were those from Lee et al. (47). After 3 h of infection at MOI 20, total RNA was isolated from *Aeromonas* cultures using TRIzol^®^ Reagent (Invitrogen, Carlsbad, CA, USA) as previously described (52). RNA quality and integrity were confirmed using NanoDrop 2000, calculating the 260/280 and 260/230 ratios. The cDNA was transcribed from RNA using iScript cDNA Synthesis Kit (Bio-Rad Laboratories, Inc., Hercules, CA, USA) according to the manufacturer’s instructions. Quantitative Real-Time PCR was performed in triplicate using Real-Power SYBR^®^ green PCR Mastermix (Applied Biosystems^®^, Waltham, MA, USA) on a StepOnePlus™ Real-Time PCR System (Applied Biosystems). Threshold cycle (CT) values were obtained to establish the relative RNA levels of the tested genes, using 16S rRNA gene as a housekeeping gene and then calculated with the 2^−ΔΔCt^ method.

### Statistical Analysis

All the experiments were performed in triplicate, and the statistical significance was determined by using Student’s two-tailed *t*-test and two-way ANOVA at p < 0.05 using the GraphPad Prism 6.0 (GraphPad Software, CA, USA).

## Results

### Presence of Virulence Factors

The presence of virulence-associated genes in these *Aeromonas* strains was screened by PCR. The distribution of the genes is summarized in **Table 3**. The most frequent virulence gene detected was *alt* (66.66%), followed by *aerA*, *ascV*, and *flaA* (45.83%); *hlyA*, *ascF*, and *ast* (41.66%); and *act* (37.5%). The less frequent virulence genes were *aexT* and *aexU* (20.83%). In contrast, *stx* gene was not detected in any of the 24 strains. In relation to the species as shown in **Table 3**, the results showed that *A. veronii* strains had a higher number of positive strains (25) for different genes, followed by *A. dhakensis* (23), *A. piscicola* (18), and *A. caviae* (15), while *A. media* (9) and *A. jandaei* (5) had a smaller number of positive strains.

**Table 3:** Distribution of virulence factors in *Aeromonas* strains.

Species	*ascF*	*ascV*	*aerA*	*hlyA*	*flaA*	*act*	*ast*	*alt*	*aexT*	*aexU*	*stx1*
***Aeromonas dhakensis***	2 (50)	2 (50)	4 (100)	2 (50)	2 (50)	2 (50)	3 (75)	4 (100)	1 (25)	1 (25)	0 (0)
***Aeromonas caviae***	2 (50)	1 (25)	1 (25)	2 (50)	3 (75)	0 (0)	2 (50)	3 (75)	1 (25)	1 (25)	0 (0)
***Aeromonas veronii***	3 (75)	3 (75)	3 (75)	3 (75)	2 (50)	3 (75)	1 (25)	3 (75)	2 (50)	2 (50)	0 (0)
***Aeromonas media***	2 (50)	1 (25)	0 (0)	1 (25)	2 (50)	0 (0)	1 (25)	2 (50)	0 (0)	0 (0)	0 (0)
***Aeromonas jandaei***	0 (0)	1 (25)	2 (50)	0 (0)	0 (0)	1 (25)	0 (0)	1 (25)	0 (0)	0 (0)	0 (0)
***Aeromonas piscicola***	1 (25)	2 (50)	1 (25)	1 (25)	2 (50)	3 (75)	3 (75)	3 (75)	1 (25)	1 (25)	0 (0)

Number (%) of positive strains.

### Intracellular Survival

**Figure 1:**
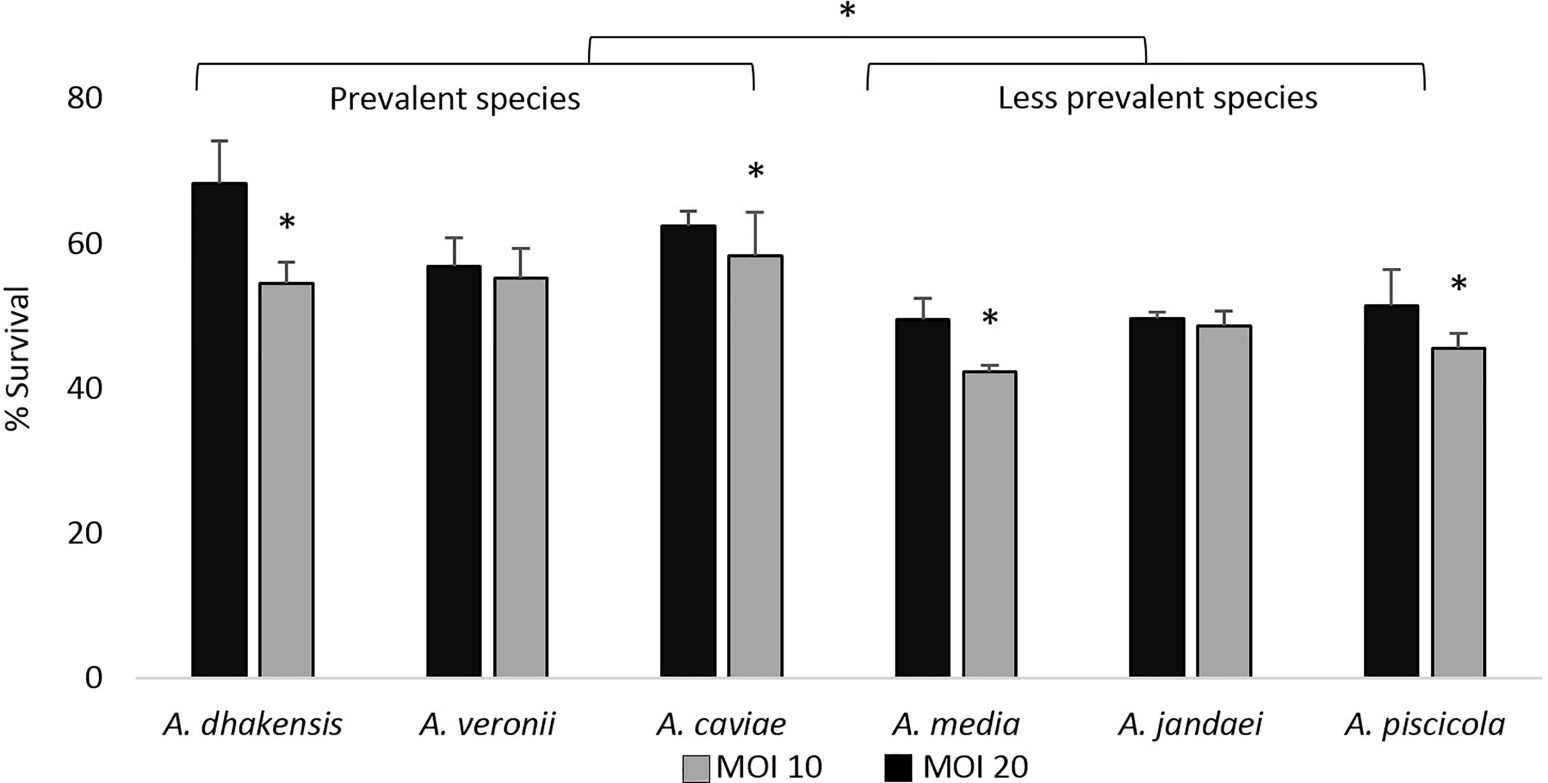
Intracellular survival expressed as the average of the strains of each *Aeromonas* species after 4 h of THP-1 monocytic cell line infection at multiplicities of infection (MOI) 10 and 20. Asterisks indicate statistical significance *p < 0.05.

As shown in **Figure 1**, independently of the MOI, the most prevalent clinical species (*A. dhakensis*, *A. veronii*, and *A. caviae*) showed, in general, a higher intracellular survival than the less prevalent species (*A. media*, *A. jandaei*, and *A. piscicola*). Additionally, at MOI 20, the intracellular survival of *Aeromonas* spp. was significantly higher (p < 0.05) than at MOI 10 except for the species *A. jandaei* and *A. veronii* (**Figure 1**).

### Cell Damage Caused by *Aeromonas* Species

**Figure 2:**
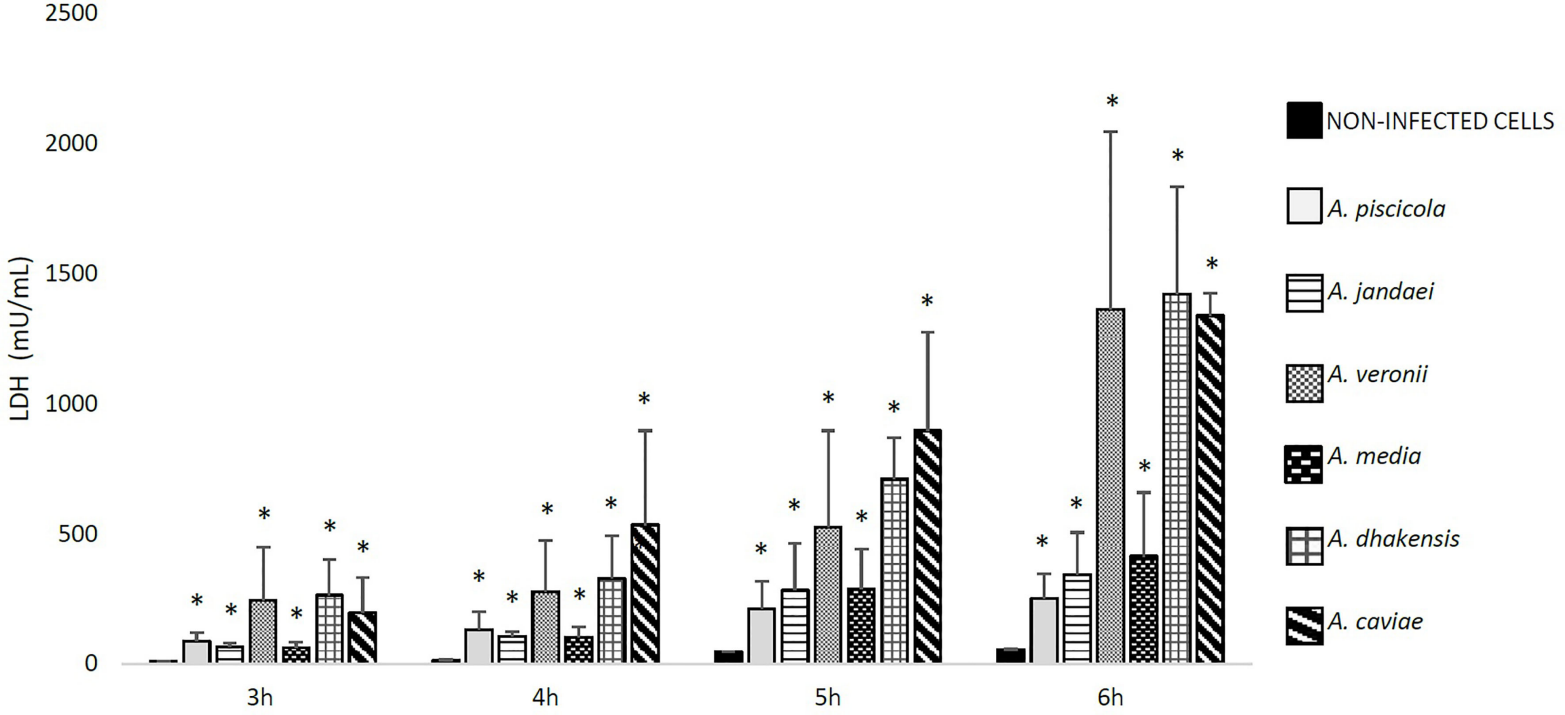
Detected THP-1 cell damage induced by the six different *Aeromonas* spp. at multiplicity of infection (MOI) 20 and at different exposure times in relation to the non-infected cells, measured by the release of lactate dehydrogenase (LDH) enzyme. Asterisks indicate statistical significance *p < 0.05.

**Figure 3:**
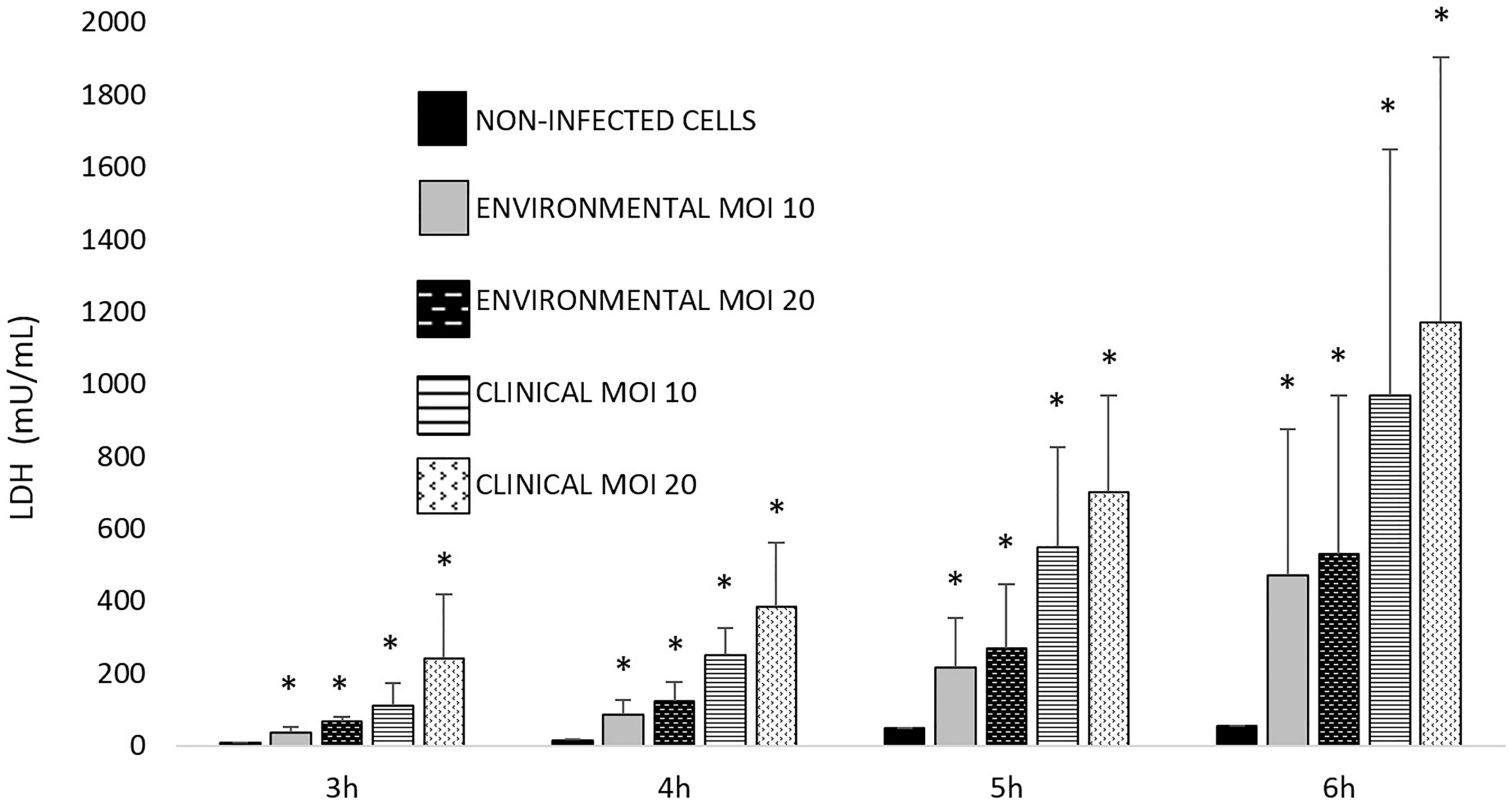
Observed THP-1 cell damage induced by clinical and environmental strains of *Aeromonas* spp. at different exposure times and multiplicities of infection (MOIs). Asterisks indicate statistical significance *p < 0.05.

The ability of *Aeromonas* to induce cell damage in THP-1 cells measured as the release of LDH to the cell culture supernatant is shown in **Figures 2**, **3**. The six *Aeromonas* species caused significantly higher cell damage (p < 0.05) when compared with the non-infected cells, and this was higher after the infection at MOI 20 (**Figure 2**). All *Aeromonas* strains, independently of the species, were able to induce at MOI 10 and 20 significant degrees of cell damage that increased with time (p < 0.05). The clinical strains of all the species were able to induce a higher degree (p < 0.05) of THP-1 cell damage than the environmental strains (**Figure 3**). In addition, the more prevalent clinical species, *A. dhakensis*, *A. caviae*, and *A. veronii*, caused significantly higher cell damage than the rest, independently of the exposure time (p < 0.05) (**Figure 2**).

### Monocytic Cell Line (THP-1) Gene Expression After *Aeromonas* spp. Infection

#### Genes Involved in Pathogen Recognition

**Figure 4:**
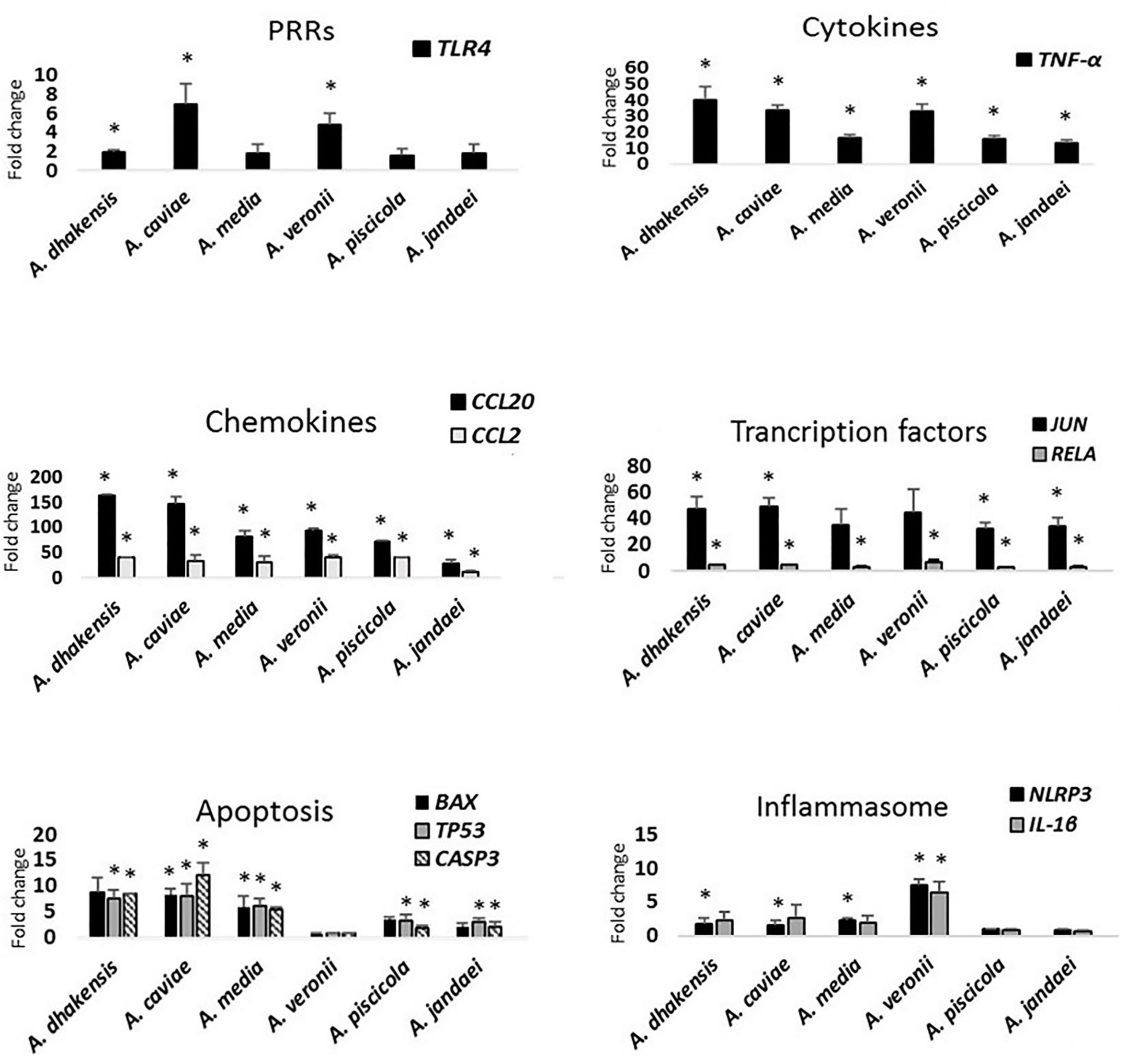
Gene expression profile of THP-1 cells in relation to the non-infected cells induced by the different studied *Aeromonas* spp. at multiplicity of infection (MOI) 20 determined by RT-qPCR. Transcript levels of the genes were normalized to the expression of *GAPDH* gene. Expression fold change with respect to the non-infected cells was calculated using the comparative ΔΔCt method. Asterisks indicate statistical significance *p < 0.05.

After the *Aeromonas* infection, the transcriptional level of *TLR4* gene encoding TLR4 showed significant differences (p < 0.05) in comparison with the expression of this gene in the non-infected cells, but this only occurred after infection with *A. dhakensis*, *A. caviae*, and *A. veronii* (**Figure 4**).

#### Genes for Cytokines and Chemokines

All *Aeromonas* strains, independently of the origin and the species, induced the THP-1 monocytic cells to express the genes that encode the cytokine TNF-α and the chemokines (CCL2 and CCL20), with a significant difference (p < 0.05) in the transcription pattern in relation to the non-infected cells (**Figure 4**). However, the expression levels of *TNF-α* gene were higher in response to the infections produced by *A. dhakensis*, followed by *A. caviae* and *A. veronii* (p < 0.05). The expression level of the pro-inflammatory cytokine IL-8 was below the detection limit for all species (**Figure S1**).

In the case of chemokines, the transcriptional levels of *CCL2* and *CCL20* genes were upregulated after infection with all strains in comparison with the non-infected cells, but a higher expression was observed for *CCL20* gene and especially for infections produced with *A. dhakensis* and *A. caviae* than with the other species (p < 0.05) (**Figure 4**). The transcriptional levels of *CCL2* and *CCL20* genes after *A. veronii* infection showed no significant differences with *A. media* or *A. piscicola*. However, all species showed a significantly higher expression of chemokines (p < 0.05) than *A. jandaei* (**Figure 4**).

#### Genes of Transcription Factors

The THP-1 cells responded by upregulating the JUN transcription factor after infection with all the *Aeromonas* spp. (p < 0.05). However, the upregulation was not significantly different from the non-infected cells in the case of *A. media* and *A. veronii* (**Figure 4**). *RELA* gene was also overexpressed (p < 0.05) in relation to the non-infected cells but at a lower level than *JUN* gene.

#### Genes Involved in Apoptosis

The transcriptional level of *BAX*, *TP53*, and *CASP3* apoptosis genes increased after infection with all *Aeromonas* species (p < 0.05), with the exception of *A. veronii*, which showed a very low expression (**Figure 4**). The expression of *BAX* gene showed no significant differences in relation to the non-infected cells after *A. dhakensis*, *A. piscicola*, and *A. jandaei* infections (**Figure 4**). The *BAX*, *TP53*, and *CASP3* apoptosis genes showed a higher expression (p < 0.05) after infection with the species *A. dhakensis*, *A. caviae*, and *A. media* in comparison with the other species (**Figure 4**).

#### Genes Related to the Inflammasome and Pyroptosis

*NLRP3* and *IL1-β* genes, which are related to pyroptosis, i.e., cell-death mediated by the formation of the inflammasome, were upregulated in THP-1 cells in response to *A. dhakensis*, *A. caviae*, *A. media*, and *A. veronii* infections (p < 0.05), while the gene expression was very low after infection with *A. piscicola* and *A. jandaei* and showed no significant differences in relation with the expression in the non-infected cells (**Figure 4**). The upregulation of *NLRP3* and *IL-1β* genes showed an eightfold increase (p < 0.05) when THP-1 cells were infected with *A. veronii* strains. No significant differences in the level of the *IL-1β* gene expression were observed when comparing the infection with *A. dhakensis*, *A. caviae*, and *A. media*. However, significant differences in the overexpression of the *NLRP3* gene were detected (p < 0.05) for the three species (**Figure 4**).

### Virulence-Associated Gene Expression

**Figure 5:**
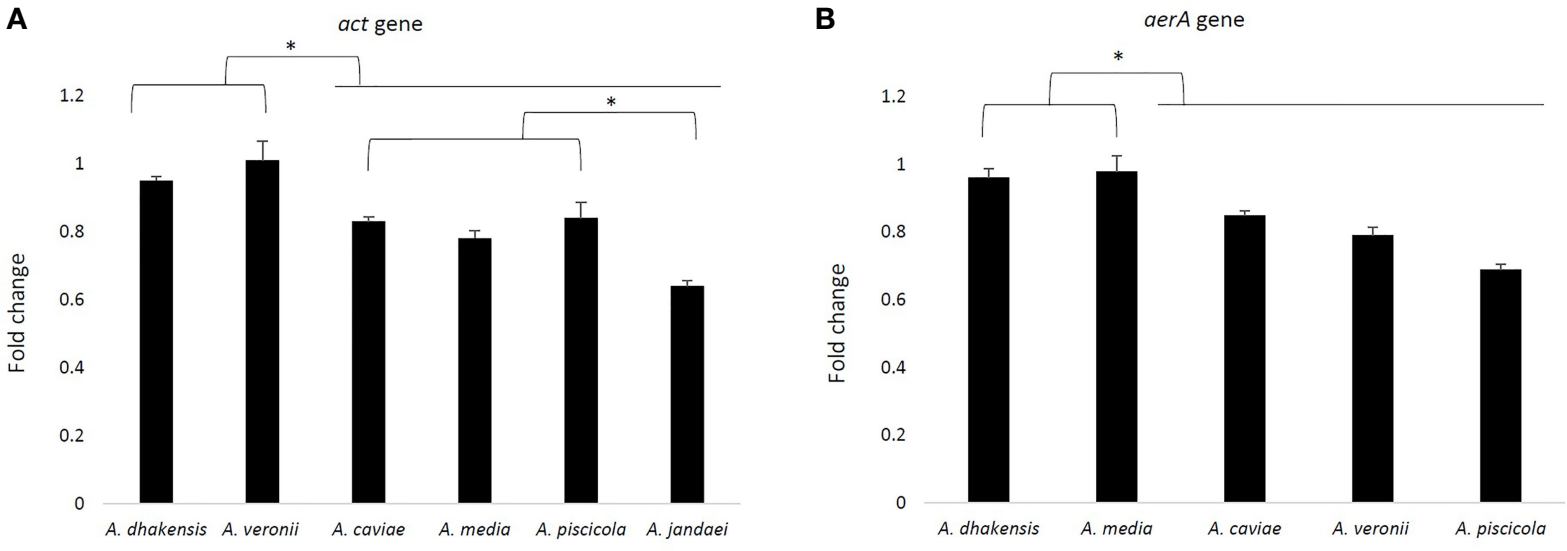
Fold change of the *act* gene expression profile of six *Aeromonas* strains (one strain for each species) **(A)** and the *aerA* gene with the exception of *A. jandaei* **(B)** after 3 h of THP-1 infection, analyzed by RT-qPCR and normalized to the reference gene 16S rRNA. Asterisks indicate a significant difference (*p > 0.05).

Gene expression of *act* and *aerA* gene is shown in **Figure 5**. The results showed a higher expression of both genes in *A. veronii* (1.01 and 0.98), followed by *A. dhakensis* (0.95 and 0.96), and significant differences with the other species after 3 h of monocyte infection (p < 0.05). The less transcriptional level of *act* gene was in *A. jandaei* (0.64) and of *aerA* gene in *A. piscicola* (0.69).

## Discussion

The TLRs are a class of PRR proteins that play a key role in the cell innate immune response to infection. In this study, the *TLR4* gene expression in THP-1 cells in response to the infections produced by different *Aeromonas* strains of *A. media*, *A. piscicola*, and *A. jandaei* was shown to be upregulated, but the expression was not significantly higher than in the non-infected cells. The TLR4 is a transmembrane receptor that recognizes a particular type of molecules from many pathogens, in the case of *Aeromonas*, the LPS, and induces the inflammatory response, producing pro-inflammatory cytokines (17, 27, 53). Previous studies with other microorganisms, including SARS-CoV-2, showed that overstimulation of the TLR4 could be detrimental leading to a hyperinflammation process called cytokine storm (54). The upregulation of the *TLR4* gene was demonstrated after *A. hydrophila* infection in catfish (27). Similarly, Srivastava et al. (55) demonstrated the activation of the TLR4 signaling pathway in zebrafish after *A. hydrophila* infection. These results suggest that *A. hydrophila* activates the TLR4 to trigger an anti-inflammatory response to facilitate their survival and pathogenesis. However, other TLR proteins such as TLR5 or TLR21 had been described after *A. hydrophila* infection by Zhang et al. (27) using fish as an infection model. For this reason, it is also possible that other TLRs, not evaluated in the present study, could be more related to the activation of the immune response after the *Aeromonas* infection with the species tested in our work. Another explanation could be related to the activation of different TLRs depending on the infected cell type studied as occurs in *V. vulnificus* between monocytes and endothelial cells (32).

Our results showed that the expression of the pro-inflammatory cytokine gene *TNF-α* was upregulated in the THP-1 monocytic cell line after infection with all the *Aeromonas* strains as also occurred for the THP-1 after *V. vulnificus* infection (32). Similar results were found after *A. hydrophila* infections of macrophages, yellowtail leukocytes, and grass carp intestinal cells (25, 56, 57). The TNF-α cytokine is involved in the inflammation, and in the absence of this protein, the host defense would be impaired (58). The overexpression of the *TNF-α* gene in the THP-1 monocytic cell line against infections produced by the six *Aeromonas* spp. tested confirms that this cytokine participates in the immunological response against *Aeromonas* infections. However, unlike results from previous studies that demonstrated the upregulated expression of the *IL-8* cytokine gene, which induces chemotaxis in neutrophils and stimulate the phagocytosis, in macrophages or in a fish model after infection with *Aeromonas* (59–61), no upregulation of *IL-8* gene in THP-1 monocytic cells could be observed in our study. An explanation of this could be associated with the varying or specific immune response of the tested infected host cell (59, 62) as it was demonstrated to occur when different cell lines were infected with *V. vulnificus* (32). In the case of the chemokine genes *CCL2*, which recruits monocytes, memory T cells, and dendritic cells, and *CCL20* chemotactic for lymphocytes and neutrophils, our data showed an upregulation of their expression in the THP-1 cells after *Aeromonas* infection. Chemokines are involved in the chemotaxis of the cells of the immune system such as monocytes/macrophages (CCL2, CCL3, CCL5, CCL7, CCL8, CCL13, CCL17, and CCL22), neutrophils (CXC chemokines), eosinophils (CCL11, CCL24, CCL26, CCL5, CCL7, CCL13, and CCL3), and T cells (CCL2, CCL1, CCL22, and CCL17), among others, participating therefore in their recruitment and the inflammation intracellular signaling control mechanisms (63). In fact, a previous study demonstrated a higher transcriptional level of the chemokine genes (*CCL2* and *CCL20*) after infection of THP-1 cells with *V. vulnificus*, confirming that monocytes played an equally important role as other immune-related cells to control the infection process (32, 35). The overexpression of *CCL2* and *CCL20* genes in the THP-1 monocytic cells after infection with all *Aeromonas* spp. suggests that monocytes play a role in inducing the recruitment of other immune-related cells.

In addition, after *Aeromonas* infection, the THP-1 cells showed an overexpression of *RELA* and *JUN* genes that encode for crucial proteins, i.e., RELA and JUN, for NF-κB activation, responsible for the expression of cytokines, which contribute to an effective immune response (29, 64, 65). Previous studies in a fish model of infection with *A. hydrophila* demonstrated the expression of *RELA* gene and the NF-κB pathway that could result in the production of several cytokines such as TNF-α, IL-1β, IL-12, IL-8, IL-6, and IFN (27, 60). In addition, the data obtained by Hayes et al. (28) suggested that JUN could be involved in the different mechanisms of virulence caused by *Aeromonas* spp. in epithelial colorectal adenocarcinoma (Caco-2) cells. Our results suggested that the overexpression of the genes that encode the transcriptional factors RELA and JUN increased the immune response against these six *Aeromonas* spp., inducing the production of cytokines and chemokines.

*NLRP3* and *IL-1β* genes related to the pyroptosis cell death mediated by the inflammasome and with an important role that triggers the immune response were clearly upregulated after *A. veronii* infection. The results of McCoy et al. (36) demonstrated that *A. hydrophila* induces an inflammatory response *via* NLRP3 inflammasome, triggers the activation of caspase-1 (CASP1), and releases IL-1β, producing pyroptosis. Additionally, another study suggested that NLRP3 and NLRC4 inflammasomes are involved in host defense against *A. veronii* infection in mice, triggering the activation of CASP1 with the consequent release of IL-1β and pyroptosis, through the action of the aerolysin and the Type 3 Secretion System (T3SS) (37). However, the expression of *NLRP3* and *IL-1β* genes after *A. jandaei* and *A. piscicola* infection of the THP-1 cells was very low and no different from the expression of the THP-1 cells without infection. An explanation for this could be the selection of a different pathway of cell death by these two species because the gene expression analysis showed an upregulation of the genes related to apoptosis (*BAX*, *TP53*, and *CASP3*). However, there was a very low expression of these genes after *A. veronii* infection. For example, in the early stage of infection, *Shigella* prevents caspase-4-dependent pyroptotic cell death by delivering the T3SS effector OspC3 (48). However, this affirmation could be related to the presence of virulence factors in each strain, such as T3SS, being therefore strain-dependent. Our results showed a similar expression of *BAX*, *TP53*, and *CASP3* genes after infection with all *A. veronii* strains (**Figures S1**-**S4**). However, in future studies, it would be interesting to analyze the genome of these strains. In the case of infections with *A. dhakensis*, *A. caviae*, and *A. media*, the THP-1 cells overexpressed the *BAX*, *TP53*, and *CASP3* genes related to the apoptosis and also those related to pyroptosis. Further studies with a higher number of strains using additional inflammasome genes, like *PYCARD*, *IL-16*, or *IL-18*, are necessary to confirm the hypothesis related to the different pathways of cell death.

The expression of immune-related genes in the present study showed that the selected species caused a different immune response, characterized by a species-specific activation pattern. Independently of the immune-related gene studied in this work, generally, the expression was higher in the most prevalent clinical species *A. dhakensis*, *A. caviae*, and *A. veronii* (5). An explanation of this could be associated with the cytokine storm, described as a systemic inflammatory response syndrome that increases the severity of the infections (66–68), and in this case, these species could induce this mechanism and the consequence could be their higher virulence and therefore prevalence in clinical cases. This is the first time that a hypothesis is provided to explain the different frequencies of occurrence of the *Aeromonas* species associated with clinical cases.

This study showed that all strains of *Aeromonas* induce the THP-1 cell damage, independently of the species (data not shown), by measuring the LDH in the supernatant. Epple et al. (69) used this technique to quantify the cell damage in epithelial colon cells, demonstrating the pathogenicity of *A. hydrophila.* The LDH release can be induced by lysing the cells by apoptosis or pyroptosis. Our results demonstrated that this cell damage could be related to both pathways, depending on the species, as previous studies suspect (36, 37). In addition, the most prevalent clinical species were shown to produce higher cell damage than the less prevalent, and this result agrees with their higher intracellular survival. Also, clinical strains of *Aeromonas* spp. produced higher cell damage than environmental strains. In the cytokine storm physiological reaction in humans, the innate immune system causes an uncontrolled and excessive release of pro-inflammatory cytokines (67). Taking into account the expression results, it could be indicated that a cytokine storm that induces a strong immune response would cause more cell damage, as well as an increase in the intracellular survival of the most prevalent clinical species *A. dhakensis*, *A. caviae*, and *A. veronii* in the THP-1 host cells. However, the presence/absence, as well as the expression levels of the virulence factors, is not associated with differences between species, since although the most prevalent species in the clinic such as *A. veronii* or *A. dhakensis* showed a large number of these, *A. piscicola* also showed in our study a large amount of virulence genes. In addition, no differences were observed between the species at the expression level of the *aerA* and *act* genes. These results coincide with those previously described in which no correlation was found between the presence/expression of virulence factors in *Aeromonas* and infection in humans (47, 70).

This is the first study that evaluates the immune response after *Aeromonas* infection using the human monocytic cell THP-1.

## Data Availability Statement

The data presented in the study are deposited in the NCBI GenBank repository, accession number: HQ442790.1; HQ442748.1; KJ743585.1; KJ743512.1; HQ442728.1; KR140072.1; HQ442833.1; FM999965.1; AY169338.1; HQ442709.1; JN711805.1; KJ743587.1; KJ743538.1; KP401037.1; KJ743556.1; KJ743508.1; KJ743508.1; HQ442840.1; HQ442859.1; HQ442690.1; KR140073.1; EU488664.1; HE965661.1; EF465510.1.

## Author Contributions

AF-B was involved in the performance of the assay, assay design, data analysis, and writing of the manuscript. MJF was involved in the conceptualization of assay, data analysis, and writing of the manuscript. All authors contributed to the article and approved the submitted version.

## Funding

The projects JPIW2013-095-C03-03 of MINECO (Spain) and AQUAVALENS of the Seventh Framework Program (FP7/2007-2013) grant agreement 311846 from the European Union supported the study.

## Conflict of Interest

The authors declare that the research was conducted in the absence of any commercial or financial relationships that could be construed as a potential conflict of interest.

## Publisher’s Note

All claims expressed in this article are solely those of the authors and do not necessarily represent those of their affiliated organizations, or those of the publisher, the editors and the reviewers. Any product that may be evaluated in this article, or claim that may be made by its manufacturer, is not guaranteed or endorsed by the publisher.
