# Supplementary material for: Immune Response of the Monocytic Cell Line THP-1 Against Six Aeromonas spp
Source: Front Immunol. 2022 Jul 8;13:875689. doi: 10.3389/fimmu.2022.875689 (PMC9304557; doi:10.3389/fimmu.2022.875689)
Supplement: Supplementary file 1 [file DataSheet_1.docx]

**Supplementary material**

**Immune response of the monocytic cell line THP-1 against six *Aeromonas* spp.**

**Ana Fernández-Bravo^1,2*^, and Maria José Figueras ^1,2^**

^1^Rovira i Virgili University, Department of Basic Medical Sciences, Mycology and Environmental Microbiology Unit, Reus, Spain.

^2^Pere Virgili Health Research Institute (IISPV), Reus, Spain

*** Correspondence:**

Ana Fernández-Bravo

[ana.fernandez@urv.cat](mailto:ana.fernandez@urv.cat)

**Keywords: *Aeromonas* spp., immune-related genes, monocytic cells, cell damage, intracellular survival.**

**Supplementary Figure 1:** *IL-8* gene expression profile of THP-1 cells in relation to the non-infected cells induced by the different studied *Aeromonas* spp. at MOI 20. The expression levels were below the detection limit for all species.

**Supplementary Figure 2:** *BAX* gene expression profile of THP-1 cells in relation to the non-infected cells induced by the four strains *A. veronii*, at MOI 20. The expression levels were below the detection limit for all species. No significant differences.

**Supplementary Figure 3:** *TP53* gene expression profile of THP-1 cells in relation to the non-infected cells induced by the four strains of *A. veronii*, at MOI 20. The expression levels were below the detection limit for all species. No significant differences.

**Supplementary Figure 4:** *CASP3* gene expression profile of THP-1 cells in relation to the non-infected cells induced *A. veronii*. at MOI 20. The expression levels were below the detection limit for all species. No significant differences.
